# A proteomic investigation of isogenic radiation resistant prostate cancer cell lines

**DOI:** 10.1002/prca.202100037

**Published:** 2021-06-30

**Authors:** Natalie Kurganovs, Hanzhi Wang, Xiaoyong Huang, Vladimir Ignatchenko, Andrew Macklin, Shahbaz Khan, Michelle R. Downes, Paul C. Boutros, Stanley K. Liu, Thomas Kislinger

**Affiliations:** 1 Princess Margaret Cancer Centre, University Health Network, Toronto, Canada; 2 Sunnybrook Research Institute, Sunnybrook Health Sciences Centre, Toronto, Canada; 3 Department of Medical Biophysics, University of Toronto, Toronto, Canada; 4 Division of Anatomic Pathology, Laboratory Medicine and Molecular Diagnostics, Sunnybrook Health Sciences Centre, Toronto, Canada; 5 Department of Laboratory Medicine and Pathobiology, University of Toronto, Toronto, Canada; 6 Departments of Human Genetics & Urology, Jonsson Comprehensive Cancer Center, Los Angeles, USA; 7 Institute for Precision Health, University of California, Los Angeles, USA

**Keywords:** CD44, DU145 cells, prostate cancer, proteomics, radiation resistance

## Abstract

To model the problem of radiation resistance in prostate cancer, cell lines mimicking a clinical course of conventionally fractionated or hypofractionated radiotherapy have been generated. Proteomic analysis of radiation resistant and radiosensitive DU145 prostate cancer cells detected 4410 proteins. Over 400 proteins were differentially expressed across both radiation resistant cell lines and pathway analysis revealed enrichment in epithelial to mesenchymal transition, glycolysis and hypoxia. From the radiation resistant protein candidates, the cell surface protein CD44 was identified in the glycolysis and epithelial to mesenchymal transition pathways and may serve as a potential therapeutic target.

## 1 ∣ INTRODUCTION

Prostate cancer (PCa) remains the most common non-skin malignancy in men, and the second leading cause of cancer related death [1]. Localised prostate cancer is stratified into risk groups based upon the local extent of the tumour (T category), grade group (GG), and prostate-specific antigen (PSA) level. Men presenting with high risk prostate cancer including local spread of cancer beyond the prostate, GG of 4 or 5, or a PSA > 20, are at considerable risk of dying from prostate cancer [2]. Surgery or radiation therapy is administered with curative intent for men with localised PCa. Traditionally, radiation therapy has been delivered to the prostate using small doses of radiation (1.8–2 Gy per fraction) daily over several weeks, a schedule referred to as conventional fractionation (CF) radiation therapy. More recently, the use of hypofractionated (HF) radiation therapy (> 2 Gy per fraction) has gained favour in the clinic. This is due to its promising outcomes, such as potentially enhanced biological effectiveness in addition to a reduced and more convenient treatment schedule [3-5]. CF and HF radiotherapy have similar rates of biochemical failure, prostate cancer specific mortality and overall survival [6]. However, cancer recurrence following prostate radiotherapy remains a significant clinical concern, particularly with high risk disease [7]. 25–50% of high risk PCa patients treated with radiation therapy will develop biochemical recurrence within 5 years following therapy, with about 20–30% succumbing to their disease within 10 years [8-10]. When PCa relapses following radiation treatment, the recurrent tumours can behave aggressively as they are generally larger and associated with lymph node metastases and have a worse prognosis [4,11]. The purpose of the study is to use an in-depth proteomic and pathway analysis to identify proteins that may serve as therapeutic targets and are enriched in radioresistant cells that were established using repetitive irradiations mimicking clinical CF and HF treatment schedules. Evaluation of the proteome from the parental compared with the CF and HF radiation resistant cells identified several common cancer pathways that were dysregulated in radiation resistance. We focused on the cell surface protein CD44 as it has been previously reported to play a role in both oncogenesis and therapy resistance. Targeting of CD44 with genetic or pharmacological approaches could radio-sensitize all three cell lines, high-lighting its potential for therapeutic gain in prostate cancer recurring following radiation therapy.

## 2 ∣ METHODS

### 2.1 ∣ Cell culture and in vitro characterization assays

Human PCa adenocarcinoma cell lines (DU145 and PC3) were purchased from the American Type Culture Collection (ATCC; VA, USA). DU145 cells were treated with 10 Gy daily for five fractions over several weeks (DU145-HF). Generation of DU145-CF, cell culture, clonogenic survival, cellular proliferation, matrigel transwell invasion, soft agar, and western blot were performed following methods previously described [12]. The generation of radiation resistant PC3 CF cells have been previously described [13, 14]. For anti-CD44 blocking antibody experiments, cells (DU145-PAR, DU145-CF, and DU145-HF) were seeded at 250 (for 0 Gy) and 4000 (for 6 Gy) cells per well in a six-well plate with 10% FBS-DMEM and treated with InVivoMaB anti-human CD44 antibody at 0 or 10 *μ*g/mL (Clone: Hermes-1, BioX-Cell, USA) in duplicates. Three hours later, cells were mock irradiated (0 Gy) or irradiated with 6 Gy dose of ionizing radiation. Cells were then placed in a humidified CO_2_ incubator at 37°C to allow colonies to form. After 10 days, colonies were stained with crystal violet (Sigma-Alderich, USA) and counted. The experiments were performed three separate times.

For CD44 siRNA knockdown experiments, cells (DU145-PAR, DU145-CF, and DU145-HF) were seeded at 3 × 10^5^ cells per well onto a six-well plate overnight. After 16 h, siRNA control or CD44 siRNA (Origene Inc., USA) were transiently transfected into cells using SiTran (Origene Inc., USA). One day later, cells were harvested and seeded at 250 and 4000 cells per well onto a six-well plate in 10% FBS DMEM in triplicates, then mock irradiated (0 Gy) or irradiated with 6 Gy dose of ionizing radiation, respectively. Cells were then placed in a humidified CO_2_ incubator at 37°C to allow colonies to form. After 10 days, colonies were stained with crystal violet (Sigma-Aldrich, USA) and counted. To confirm knockdown, the transfected cells were lysed at 48 h post-transfection and western blotting performed using anti-CD44 antibody (Cell Signaling Technology, USA). The experiments were performed three separate times.

### 2.2 ∣ Flow cytometry

DU145-PAR, DU145-CF, DU145-HF, PC3-PAR, and PC3-CF cells (1 × 10^6^) were washed three times with Stain Buffer (BD, New Jersey, USA). Cells were then resuspended in 100 *μ*l volume and stained with FITC anti-CD44 (Biolegend, San Diego, USA) or FITC anti-IgG1 isotype control (Biolegend, San Diego, USA) on ice in the dark for 30 min. Flow cytometry was performed using LSR II system (BD, New Jersey, USA) and flow cytometry analysis was done using FlowJo (BD, New Jersey).

### 2.3 ∣ Cell lysis and sample preparation for proteomic analysis

Cells were grown to ~80% confluency in 150 mm dishes (Sarstedt, Germany), washed three times with cold phosphate buffered saline (pH 7.4) before cells were pelleted. Cell pellets were resuspended in 150*μ*L of 50% (v/v) 2,2,2,-Trifluoroethanol and sample preparation was as previously described [15]. Liquid chromatography was directly coupled to an Orbitrap Fusion Tribrid (Thermo Scientific). Data was acquired in positive-ion data-dependent mode. MS1 data was acquired at a resolution of 240,000 in the orbitrap with maximum injection time (maxIT) of 50 ms and 40s dynamic exclusion, while MS2 was acquired in the ion trap at ‘Normal’ scan rate, maxIT of 4 ms HCF fragmentation was done at a normalised collision energy of 31% and the S-lens RF was set to 60°. RAW Files were searched against a Uniprot human sequence database and the Andromeda algorithm as part of MaxQuant software [16] with an false discovery rate (FDR) set to 1% for peptide spectral matches and protein identification using a target-decoy strategy [17]. Searches were performed with oxidation of methionine residues as a variable modification, the carbamidomethylation of cysteine residues as a fixed modification, as well as a maximum of two missed cleavages. iBAQ matching, matching between runs within a 2-min retention time window, as well as MaxLFQ were enabled to perform label-free quantitation. The ProteinGroup.txt file was used for all subsequent analysis and proteins identified with two or more peptides were carried forward, and protein contaminants removed. Relative quantification was performed using iBAQ values.

### 2.4 ∣ Consensus clustering of proteomic data

Consensus clustering was performed using ConsensusClusterPlus [18] (v1.5.0) on the median normalised log_2_ iBAQ values of the total number of proteins (4410) identified across the whole cell lysates from all cell lines, in triplicate. Parameters used for consensus clustering were; max_k = 7, Euclidean P as the similarity metric, pItem = 0.8, seed = 1000, and reps = 1000. For visualisation, abundances were converted to z-scores.

### 2.5 ∣ Pathway analysis

Proteins of interest were processed for pathway analysis using the non-ranked method g:Profiler [19] (e100_eg47_p14_7733820, database updated on 07/07/2020). The whole cell lysate data was searched using an ordered query of fold change, with the list of all proteins detected as the background, and with the Molecular Signature Database 50 Hallmarks of Cancer Gene List as a reference. For the radiation enriched pathway analysis, proteins of interest were searched against the Gene Ontology terms in g:Profiler using an ordered query based on significance, and subsequently visualised in Cytoscape (v 3.7.2) [20] using the Enrichment Map App [21]. Grouping of similar pathways was created manually using Inkscape (v0.92.3; https://www.inkscape.org). g:Profiler was run on the enriched radiation lists separately, however visualised in the same instance to enable better visualisation of protein overlaps in pathways.

### 2.6 ∣ Quantification and statistical analysis

The specific statistical tests used are indicated in the figure legends and were performed using the R statistical environment (v3.6.3) (R Core Team, 2020). ggPlot2 (3.2.1) [22], ComplexHeatmap (v2.2.2) [23] packages were used for visualisation in R. For the in vitro experiments, statistical analyses were performed using Prism Graphpad 6 (Graph-Pad Software, USA) and *P*-values less that 0.05 were considered as significant.

## 3 ∣ RESULTS AND DISCUSSION

### 3.1 ∣ Creation of radiation resistant cell lines

While radiation therapy is an effective treatment modality for many PCa patients, the development of biochemical recurrence due to radiation resistance remains a clinical concern. Recent advances in the clinic have enabled the variation in radiation schedules with the two predominant ones being investigated in this paper using a cell line model; conventional fractionation (CF) radiation therapy and hypofractionated (HF) radiation therapy. To mimic the clinical scenario of resistance to both treatments, DU145 PCa cells were mock irradiated with 0 Gy (DU145-PAR), irradiated with 2 Gy daily for 59 fractions over several weeks (DU145-CF) as previously described [12] or 10 Gy daily for 5 fractions over several weeks (DU145-HF). We have previously published the data on DU145-CF cells [12] which has been presented here as an important comparator to the DU145-HF cells.

### 3.2 ∣ Conventional fractionation radiation resistance produces a more aggressive phenotype versus hypofractionation

**FIGURE 1:**
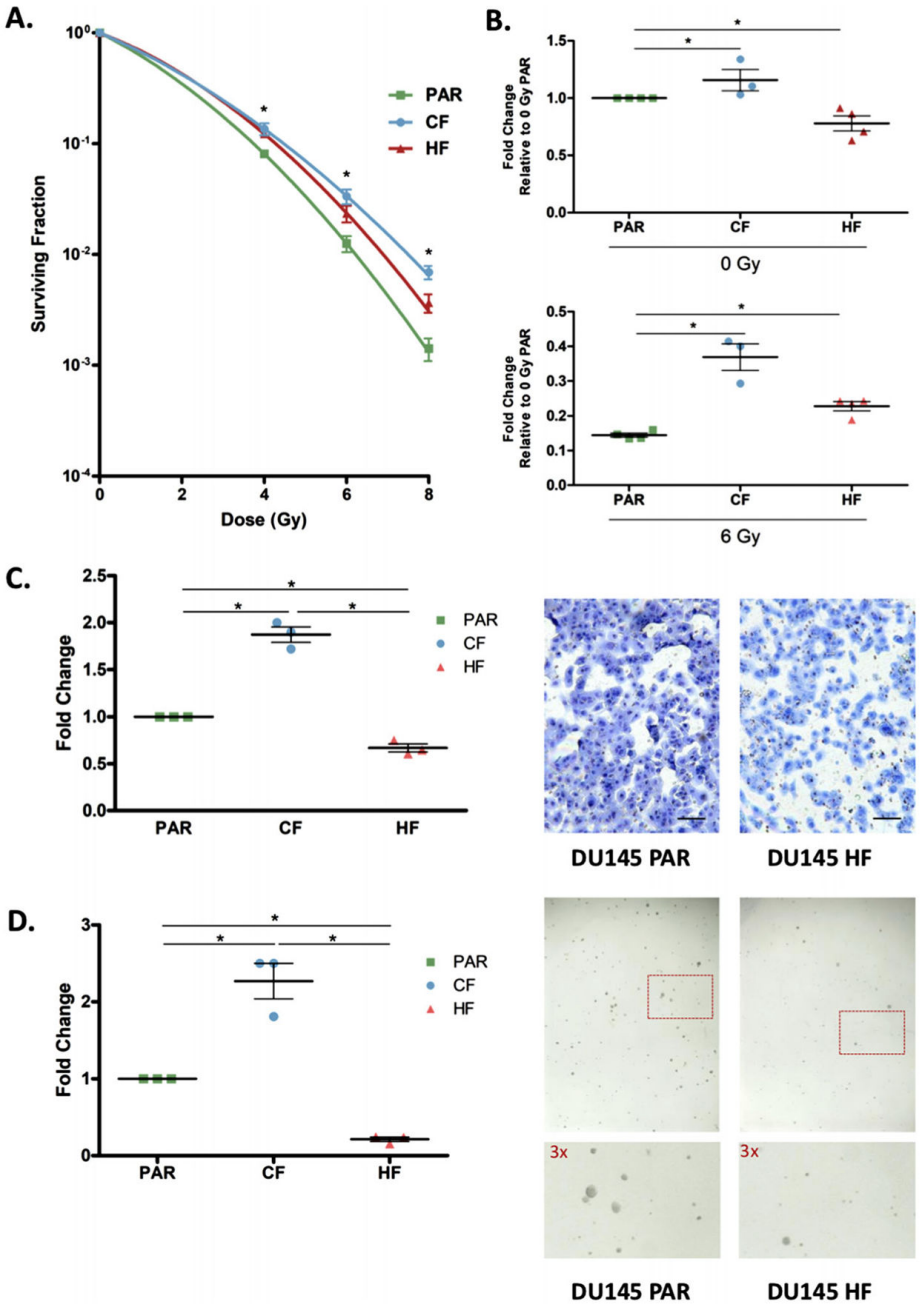
Functional analysis of DU145 cells following radiation treatment. A. DU145 cells were mock irradiated with 0 Gy (DU145 PAR), 2 Gy x 59 (DU145 CF), and 10 Gy x 5 (DU145 HF) fractionations of irradiation to generate radioresistant cells. Clonogenic survival assays were performed to assess for survival post irradiation, and the surviving fraction was fitted to the linear-quadratic equation (*N* = 3). Data are expressed as mean ± standard error of the mean. Statistical analyses were performed using Student’s t-test. *p* value < 0.05 was considered to be statistically significant. B. Fold change of viable DU145 PAR, DU145 CF and DU145 HF cells at 4 days after mock irradiation (0 Gy) or 6 Gy dose of irradiation normalized to 0 Gy PAR viability. Three or four biological replicates (3 technical replicates each) were performed and each point on the dot plot is representative of a separate biological replicate. Data are expressed as mean ± standard error of the mean. Statistical analyses were performed using Student’s t-test. *p* value < 0.05 was considered to be statistically significant. C. Matrigel transwell invasion assay of DU145 PAR, DU145 CF and DU145 HF cells. Cells were stained by eosin and methylene blue and counted. Fold change of DU145 CF and HF cells compared DU145 PAR cells are shown. Three biological replicates were performed and each point on the dot plot is representative of a separate biological replicate. A representative invasion assay is shown out of three experiments (scale bar denotes 500 *μ*m). Data are expressed as mean ± standard error of the mean. Statistical analyses were performed using ANOVA. *p* value < 0.05 was considered to be statistically significant. D. Soft agar colony formation assay of DU145 PAR, DU145 CF, and DU145 HF cells. Fold change of DU145 CF and HF colonies (> 50 cells) compared to DU145 PAR colonies are shown. Three biological replicates (3 technical replicates each) were performed and each point on the dot plot is representative of a separate biological replicate. A representative colony formation assay is shown out of three experiments with a section of each well shown at higher magnification. Data are expressed as mean ± standard error of the mean. Statistical analyses were performed using ANOVA. *p* value < 0.05 was considered to be statistically significant

Clonogenic survival assays indicated that DU145-CF cells were significantly more resistant to acute exposure of irradiation compared to DU145-PAR cells while the DU145-HF cells were radiation resistant but exhibited less resistance relative to CF cells (t-test; DU145-CF: *p* = 0.0029 for 4 Gy, *p* = 0.0015 for 6 Gy, *p* = 0.0001 for 8 Gy; DU145-HF: *p* = 0.0011 for 4 Gy, *p* = 0.0242 for 6 Gy, *p* = 0.0083 for 8 Gy; Figure 1A), suggesting that the radiation treatment schedule used can have an important impact on the resulting phenotype. Proliferation plays a vital role in both the development and progression of cancer cells. DU145-CF cells proliferated at a higher rate compared to DU145-PAR cells (t-test; *p* = 0.01 for 0 Gy and *p* = 0.02 for 6 Gy, Figure 1B) whereas DU145-HF cells initially proliferated at a lower rate than DU145-PAR cells under mock irradiation (t-test; *p* = 0.0156), and proliferation increased following 6 Gy irradiation (t-test; *p* = 0.0011; Figure 1B). A key factor for an aggressive phenotype in cancer is invasiveness, which increases the predisposition for regional lymphatic and distant metastatic spread, and may have enrichment in radiation-resistant cancers [13]. Matrigel transwell assays showed that DU145-CF cells had a greater invasive potential than DU145-PAR cells (ANOVA; *p* < 0.0001; Figure 1C), while DU145-HF cells had a lower invasive potential compared to DU145-PAR cells (ANOVA; *p* < 0.0001; Figure 1C). Cellular growth and transformation is strongly correlated to tumorigenicity in animals, and the soft agar colony formation assay was used to evaluate anchorage-independent cell growth [24]. Tumorigenic potential was significantly enhanced in DU145-CF cells compared with DU145-PAR (ANOVA; *p* = 0.0001; Figure 1D); however, it was decreased in DU145-HF cells compared with DU145-PAR (ANOVA; *p* = 0.0001, Figure 1D).

Interestingly, DU145-CF demonstrated a far more aggressive phenotype overall when compared to DU145-HF. This is consistent with previous studies which have shown hypofractionation may lead to superior outcomes for local control and distant metastasis in comparison to conventional fractionation [25,26]. These radiation resistant cell lines may reflect the clinical setting of recurrent disease, with commonalities and differences between radiation resistance emerging from these two clinical treatment regimes.

### 3.3 ∣ The proteome of radiation resistant prostate cancer cells

**FIGURE 2:**
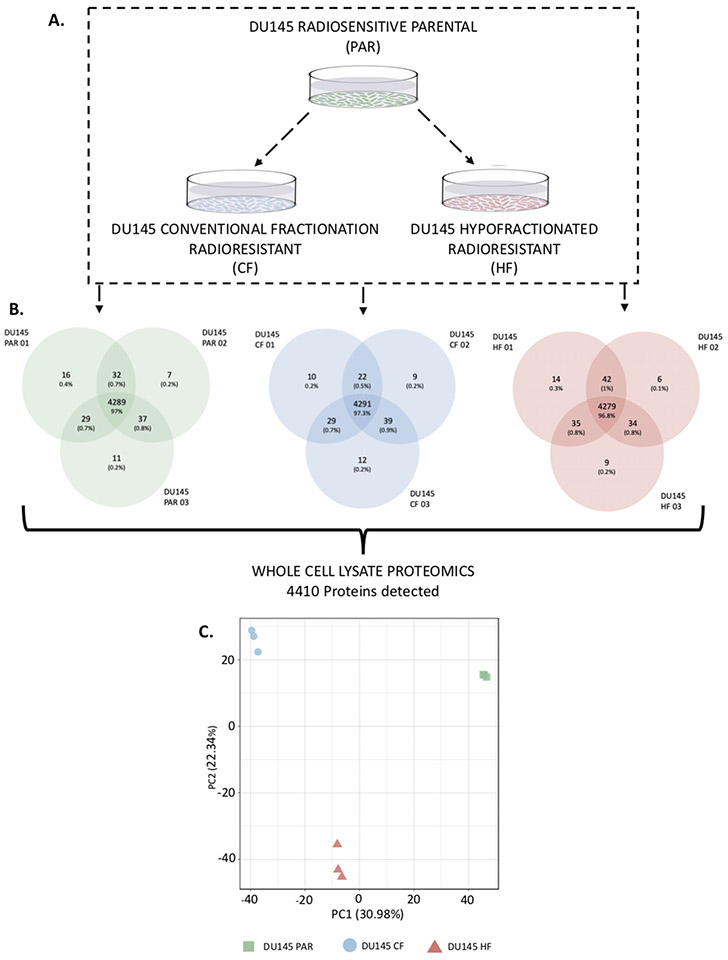
Overview of proteomic analysis performed on DU145 PAR, DU145 CF, and DU145 HF cells. A. An outline of the experimental approach to investigate the effect of radiation on DU145 cells. Whole cell lysate proteomics was performed on DU145 PAR, DU145 CF, and DU145 HF cells and a total of 4410 proteins were detected. Biological triplicates were used for each sample. B. Average protein counts detected. C. Principal Component Analysis (PCA) of whole cell lysate that characterizes the trends exhibited by the proteomic profiles of DU145-PAR (green), DU145-CF (blue), and DU145-HF (red) in triplicate. Each dot represents a sample and each colour is representative of the sample type.

**FIGURE 3:**
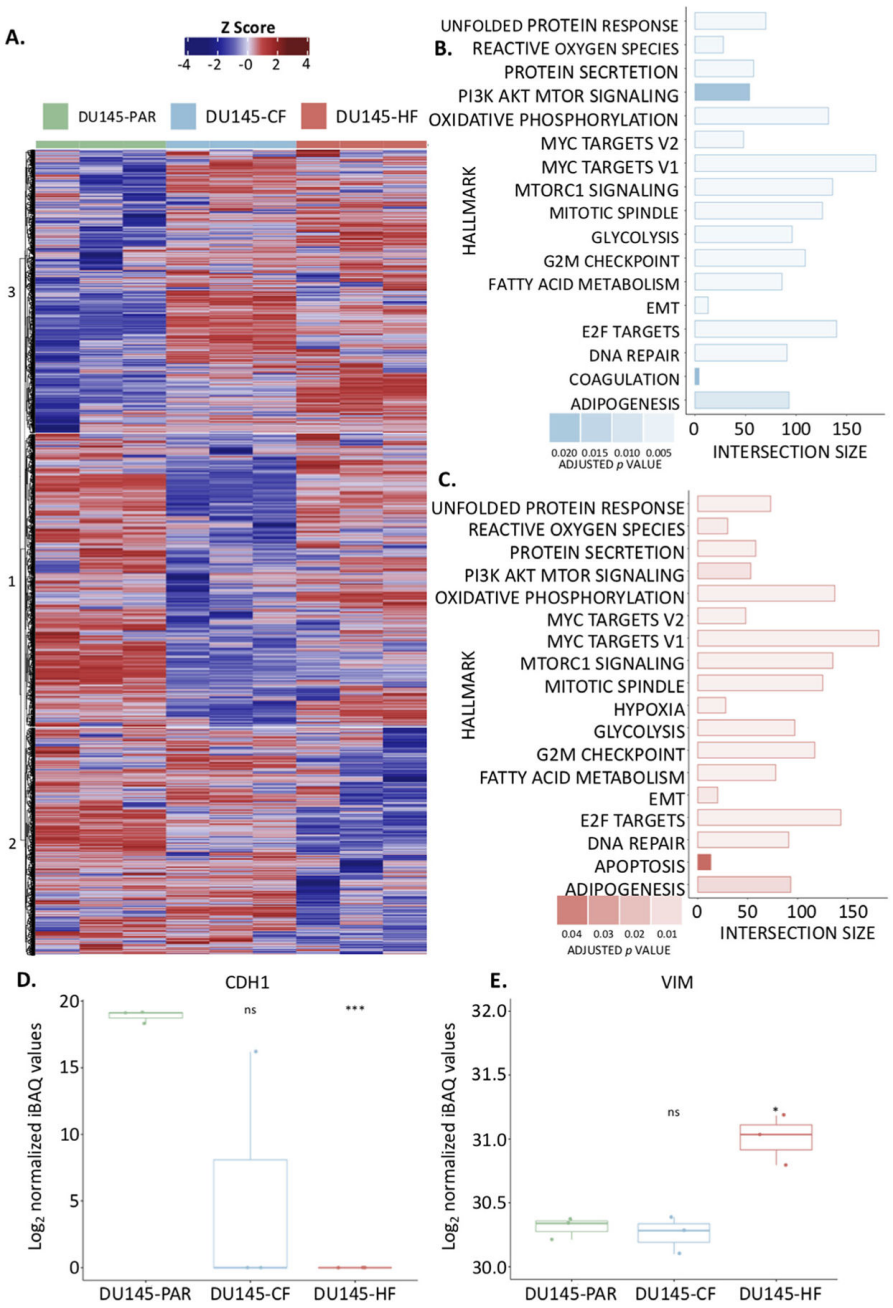
Proteomic investigation of radiation resistant cell lines. A. Heatmap showing the z-scores calculated from the relative protein abundance (log2 iBAQ values) for each of the 4410 proteins identified in the whole cell lysates for DU145-PAR (green), DU145-CF (blue), and DU145-HF (red) cells. Hard clustering was performed on this data with an ideal cluster of three identified. B. g:Profiler results show the intersection size of the proteins detected with the proteins in each pathway from the MsigDB Hallmarks of Cancer Gene List observed in the DU145 CF cell line as compared to the DU145 PAR cell line. C. g:Profiler results show the intersection size of the proteins detected with the proteins in each pathway from the MsigDB Hallmarks of Cancer Gene List observed in the DU145 HF cell line as compared to the DU145 PAR cell line. D.The normalized log2 values for E-cadherin (CDH1) across all replicates in all three cell lines. Statistical analyses were performed using Student’s t-test. *p* value < 0.05 was considered to be statistically significant. E. The normalized log2 values for Vimentin (VIM) across all replicates in all three cell lines. Statistical analyses were performed using Student’s t-test. *p* value < 0.05 was considered to be statistically significant.

To investigate both the similarities and differences observed between the radiation resistant cell lines and the parental cell lines, the proteome of the whole cell lysates was evaluated (Figure 2A). A total of 4432 protein groups were detected across the three cell lines whole cell lysates in biological triplicate (obtained from three separate whole cell lysates) (Figure 2B), with principle component analysis (Figure 2C) showing a clear proteomic separation of these cell lines. The iBAQ values for each sample were median normalised, the data was further filtered to remove proteins which were found in fewer than three samples (across all samples), leaving a total of 4410 proteins across all samples (Table S1). Consensus clustering using ConsensusClusterPlus [18] with Euclidean P as the similarity metric was used to cluster the log_2_ median normalised iBAQ values (converted to z-scores) from the 4410 proteins detected across the cell lines (Figure 3A). An optimal km of 3 was found, which clustered the samples based on the three cell lines. The 50 Hallmarks of Cancer Gene Lists from the Molecular Signatures Database (MSigDB) were compared against the proteins identified and eight pathways of interest were identified which are related to PCa (Figure S1, radiation resistance or contained a high number of proteins detected in the whole cell lysate data in either radiation resistant cell line; and these pathways were used to annotate the heatmap (Figure 3A). A total of 295 proteins were found to have a significant change in expression in DU145-CF cells as compared to the DU145-PAR cells based on a *p*-value < 0.05 and a fold change > 1 or ← 1 (130 proteins upregulated, 165 proteins downregulated; Table S1). The DU145-HF cell line had less proteins with a significant change in expression with 194 proteins at a *p*-value < 0.05 and a fold change > 1 or ← 1 (107 proteins upregulated; 87 proteins downregulated; Table S1).

### 3.4 ∣ Pathways dysregulated in radiation resistant cells

Radiation induces DNA double stranded breaks which can result in cell death [27]. A myriad of pathways, however, may be exploited by cancer cells to persist radiation. To investigate which pathways are aberrated in each cell line, protein intensities from each radiation resistant cell line (DU145-CF and DU145-HF) were compared against that from the DU145-PAR cell line for pathway analysis using g:Profiler [19], ordered based on fold change (with missing values as NA), using the 50 Hallmarks of Cancer Gene Lists from MSigDB (Figure 3B and 3C, respectively). 17 pathways were found to be enriched in the DU145-CF cells compared to DU145-PAR (Figure 3B) and 18 pathways were enriched in the DU145-HF cells compared to the DU145-PAR (Figure 3C) (Table S2). Similar pathways were enriched across both radiation resistant cell lines, including DNA repair, E2F targets, EMT, glycolysis, oxidative phosphorylation, PI3K AkT mTOR signaling and reactive oxygen species. The pathways found to be uniquely enriched in one of the radiation resistant cell lines were coagulation in the DU145-CF cells compared to DU145-PAR cells and apoptosis and hypoxia in the DU145-HF cells as compared to DU145-PAR cells.

We then analyzed the MSigDB 50 Hallmarks of Cancer Gene Lists for eight pathways implicated in radiation resistance (DNA repair, E2F targets, EMT, glycolysis, hypoxia, oxidative phosphorylation, PI3K AkT mTOR signaling and reactive oxygen species). These gene lists were compared to the significantly altered proteins (295 proteins for DU145-CF cells and 194 for DU145-HF cells) of both radiation resistant cell lines, and presented as the z-scores of the log_2_ median normalised values in a stacked heatmap in Figure S2A predominant trend towards an upregulation of proteins in EMT, glycolysis and hypoxia pathways were observed in the resistant cells as compared to the parental cell line. EMT has previously been linked to radiation resistance, chemoresistance and cancer stem cell populations in PCa [28-30]. It is a reversible cellular state that places epithelial cells transiently into quasi-mesenchymal states [31] characterised by the loss of apical-basal polarity, cellular adhesion molecules and cell-cell junctions of involved epithelium [32]. This is observed through the loss of epithelial morphology markers (such as E-cadherin (CDH1) [33, 34] and the gain of mesenchymal morphology markers (such as vimentin (VIM)[35]), with a loss of CDH1 being a hallmark of EMT. This loss has been found to contribute to the radioresistance of cancer cells through its link to an impairment of radiation-induced DNA damage during hypoxia [36]. Previously, it has been shown to have a crucial role in the aggressiveness of PCa [37, 38]. The loss of CDH1 has been found to contribute to the radioresistance of cancer cells through its link to an impairment of radiation-induced DNA damage during hypoxia [36]. Although CDH1 and VIM are not included in the MSigDB 50 Hallmarks of Cancer EMT signature; CDH1 was found to be undetected in DU145-HF cells, and lost in all but one replicate of the DU145-CF cell line (Figure 3D); while VIM was found to have a significant increase in DU145-HF cells alone (Figure 3E). This indicates the DU145-HF cells appear to have a more mesenchymal phenotype compared to the DU145-CF and DU145-PAR cells. DU145-CF cells also appear to have a more mesenchymal phenotype as compared to the DU145-PAR, however this is not as strong as that seen in the DU145-HF cells.

Oxygen plays a key role in the response of irradiation-induced reactive oxygen species, a major problem with radiation therapy is hypoxia [39]. It has been previously shown that cancer cells in a hypoxic environment are more likely to survive and proliferate compared to cancer cells in a normoxic environment [40]. Hypoxia is an important regulator of tumour growth and has long been considered to have a vital role in resistance to radiation therapy [41] due to hypoxia activating a diverse group of genes and related pathways which support an adaptation to stress and survival [42, 43]. While a trend towards upregulation in hypoxia was observed in DU145-CF cells, it was only found to be significantly altered in DU145-HF cells.

Most cancer cells display an increase in aerobic glycolysis and use this metabolic pathway for the generation of ATP as the main source of energy [44]. This increase may be regarded as a cellular adaptation to hypoxia which will lead to an elevation in lactate production which, in turn, leads to acidification of tumour tissue providing a microenvironment promoting and selecting cells with malignant behaviours [45]. Due to the mitochondria being an energy-generation organelle, mitochondrial dysfunction as a result of radiation would mediate alterations or adaptive response of metabolic pathways, such as oxidative phosphorylation, glycolysis and reactive oxygen species [46, 47], which are involved in the development of radiation resistance [48-50]. Irradiation can also induce mitochondrial dysfunction including a decrease in electron transport chain complex activities leading to persistent oxidative stress [51]. These pathways were all observed to be significantly altered in the radiation resistant cell lines. This further supports the importance of these pathways in promoting resistance to radiation therapy, regardless of the treatment modality.

### 3.5 ∣ Radiation resistant enriched proteins

**FIGURE 4:**
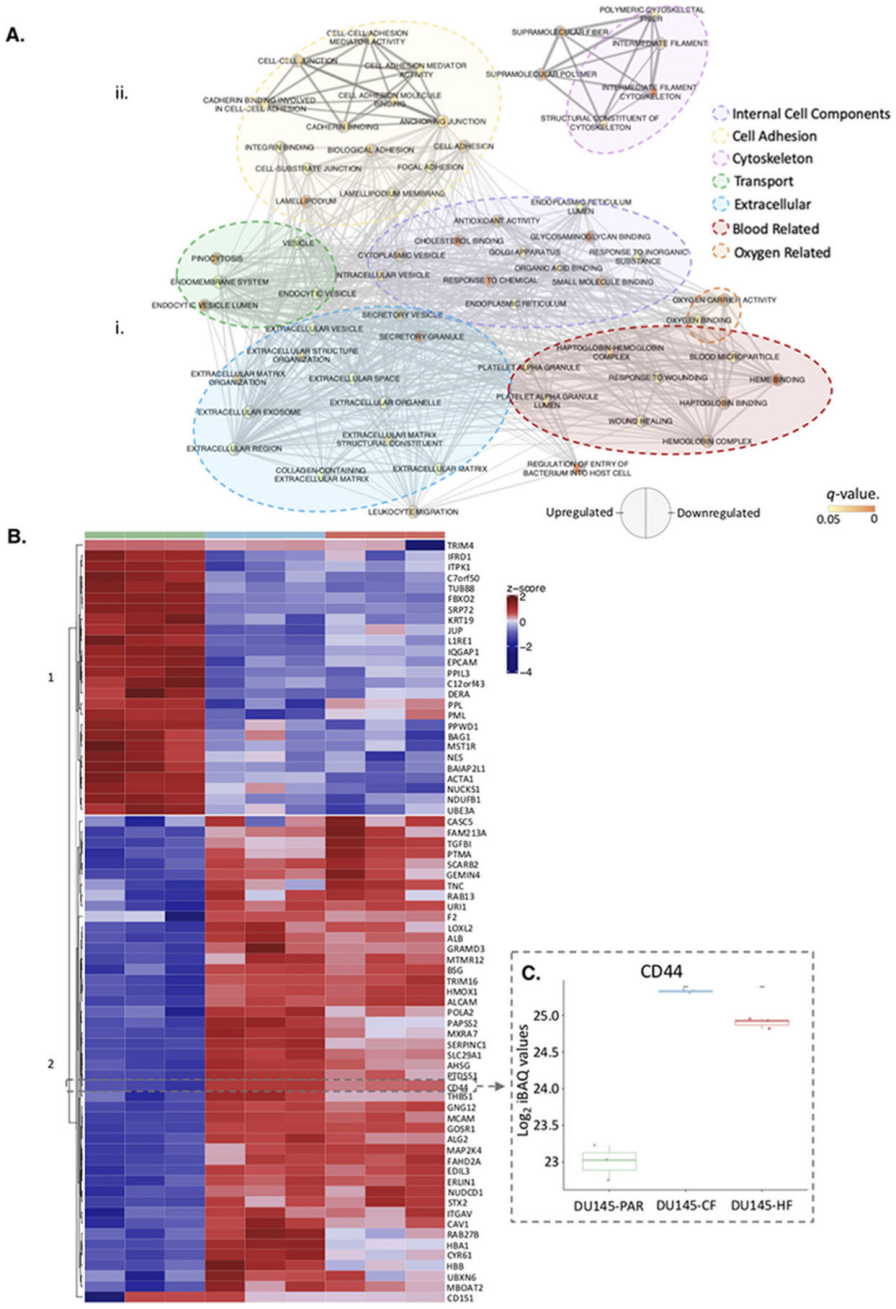
Proteins identified to be enriched in radiation resistance. A. Pathway enrichment analysis performed using g:Profiler on the (i) significantly downregulated and (ii) significantly upregulated proteins in Figure S3 Large clusters of similar pathways are outlined as internal cell components (purple), cell adhesion (yellow), cytoskeleton (pink), transport (green), extracellular (blue), blood related (red) and oxygen related (orange). B. Heatmap showing the 72 radiation resistant proteins which had a significant change in expression (represented as circles in (Figure S3. Hard clustering was applied, with an optimum km = 2 identified. CD44, a protein of interest, has been manually highlighted. C. Log_2_ normalized iBAQ values for CD44 in DU145-PAR (green), DU145-CF (blue), and DU145-HF (red). Statistics were performed using Student’s t-test. *p* values < 0.05 was considered statistically significant

We were interested in investigating proteins dysregulated by radiation, regardless of fractionation since they could represent common targets to tackle radiation resistance. In comparison to the radiation sensitive DU145-PAR cell line, the fold change of the 4410 proteins identified across all cells was compared (each radiation resistant cell line compared to the DU145-PAR cells; Figure S3 . A R^2^ of 0.3521269 was calculated between the fold change in DU145-CF cells (as compared to DU145-PAR) and the DU145-HF cells (as compared to DU145-PAR), indicating there is a low correlation observed in the fold changes of radiation resistant cells. There were 72 proteins (Table S3) which had a significant fold change (*p* < 0.05, fold change > 1 or ← 1) in both cell lines, as represented in the plot as circles. The significantly altered proteins have been highlighted (Figure S3A), and the proteins within each group (27 downregulated and 45 upregulated) were run separately on g:Profiler(searching Gene Ontology gene lists only) to determine the enriched pathways. The output from the g:Profiler search, showing the enriched pathways from the 72 proteins significantly altered in both cell lines, was combined and visualized in Cytoscape (Figure 4A). The identified gene ontology pathways were grouped based on similar functions. From the significantly altered proteins; the downregulated proteins were related to *internal cell components* and *cell adhesion* while the upregulated proteins were related to *cell adhesion, cytoskeleton, transport, extracellular, transport, blood related,* and *oxygen related.*

### 3.6 ∣ CD44 is enriched in radiation resistant cells and is a potential therapeutic target

**FIGURE 5:**
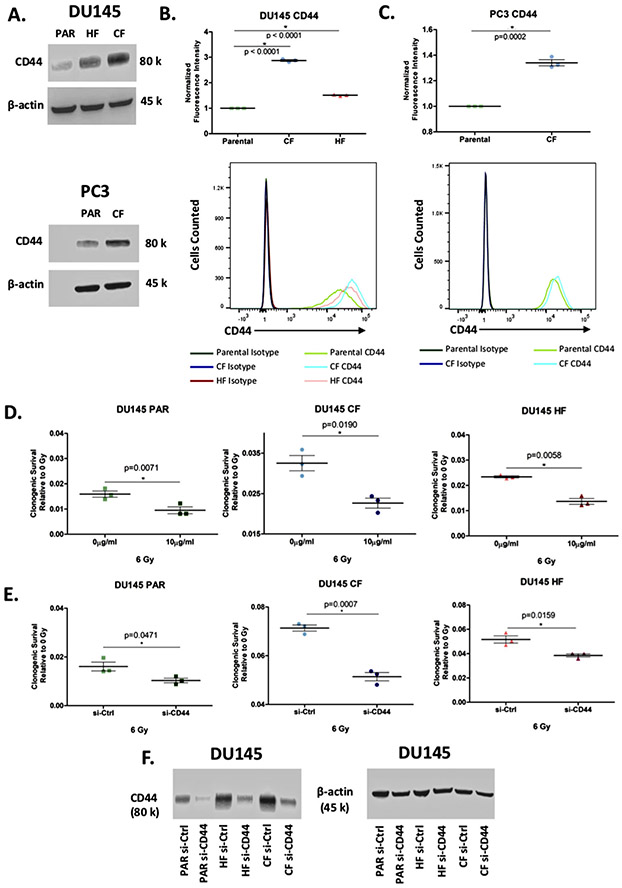
Expression of CD44 is linked to radiation resistance. A. Western blot showing the expression of CD44 in whole cell lysates from DU145-PAR, DU145-CF, DU145-HF, PC3-PAR, and PC3-CF with *β*-actin as a loading control. B. Flow cytometry analysis of surface expression of CD44 on DU145-PAR, DU145-CF, and DU145-HF (*N* = 3). Geometric mean of FITC-CD44 fluorescence intensity is normalised to isotype control A representative replicate of CD44 fluorescence intensity compared to isotype control is shown. Data are expressed as mean ± standard error of the mean. Statistical analyses were performed using Student’s t-test. *p* value < 0.05 was considered to be statistically significant. (C) Flow cytometry analysis of surface expression of CD44 on PC3-PAR and PC3-CF (*N* = 3). Geometric mean of FITC-CD44 fluorescence intensity is normalised to isotype control. A representative replicate of CD44 fluorescence intensity compared to isotype control is shown. Data are expressed as mean ± standard error of the mean. Statistical analyses were performed using Student’s t-test. *p* value < 0.05 was considered to be statistically significant. D. DU145 PAR (green), DU145 CF (blue), and DU145 HF (red) treated with 0 Gy or 6 Gy irradiation in combination with 0 *μ*g/mL or 10 *μ*g/mL anti-CD44 monoclonal antibody (mAb) (*N* = 3). Clonogenic survival of each treatment is normalized to 0 Gy + 0 *μ*g/mL mAb. Data was expressed as mean ± standard error of the mean. Statistical analyses were performed using Student’s t-test. *p* value < 0.05 was considered to be statistically significant. E. DU145-PAR (green), DU145-CF (blue), and DU145-HF (red) treated with 0 Gy or 6 Gy irradiation in combination with CD44 siRNA or control siRNA (n = 3). Clonogenic survival of each treatment is normalized to 0 Gy + control siRNA. Data was expressed as mean ± standard error of the mean. Statistical analyses were performed using Student’s t-test. *p* value < 0.05 was considered to be statistically significant. F. Western blot showing knockdown of CD44 in whole cell lysates from DU145-PAR, DU145-CF, and DU145 with *β*-actin as a loading control.

From the list of 72 radiation resistant protein candidates, CD44 was identified in the glycolysis and epithelial to mesenchymal transition pathways and selected for further investigation (Figure 4B). CD44 is a cell surface glycoprotein known to be involved in cell-cell interactions, cell adhesion and migration. It is involved in EMT and is upregulated in cancer stem cells, which can drive tumour progression and therapeutic resistance [52, 53]. Previous links of CD44 and radiation resistance have been suggested in numerous cancers including breast [54], larynx [55], and prostate [56]. The log_2_ median normalised iBAQ values for each cell line shows a significant enrichment in both radiation resistant cell lines for CD44, with DU145-CF having the highest expression (Figure 4C). Western blot was performed using DU145 and an additional PCa cell line PC3 (PC3-PAR), which also has a radio-resistant derivative (PC3-CF) generated using the same method as DU145-CF. Similar to the proteomics, total CD44 expression increased in DU145-CF, DU145-HF, and PC3-CF compared to DU145-PAR and PC3-PAR, respectively (Figure 5A). Flow cytometry confirmed increased cell surface expression of CD44 in DU145-CF, DU145-HF, and PC3-CF relative to DU145-PAR (Figure 5B) and PC3-PAR (Figure 5C). To address the therapeutic potential of CD44 targeting, significant radio-sensitisation was observed in DU145-PAR, DU145-CF, and DU145-HF treated with anti-CD44 blocking antibody combined with 6 Gy irradiation (Figure 5D). Consistent with previous studies [56, 57], knockdown of CD44with siRNA promoted radio-sensitisation in DU145-PAR as well as DU145-CF and DU145-HF (Figure 5E). Thus, targeting CD44 was able to sensitize radioresistant DU145 cells irrespective of the radiotherapy fractionation schedule employed. Xiao *et al.* also previously demonstrated targeting of CD44 with siRNA in PC3 cells promoted radiosensitivity [56]. Additionally, Dubrovska and colleagues reported that CD44 surface expression (a putative stem cell marker) was increased in radioresistant CD145, PC3, and LNCaP cells by flow cytometry (and by gene array for DU145) [58]. Together, this supports a role for CD44 in prostate cancer radioresistance.

## 4 ∣ CONCLUDING REMARKS

We were interested in identifying proteins that may contribute towards the acquisition of radiation resistance in PCa, using cell lines treated with clinically-relevant radiation schedules. We identified several dysregulated pathways including DNA repair, E2F targets, EMT, glycolysis, hypoxia, oxidative phosphorylation, PI3K AkT mTOR signaling and reactive oxygen species pathways. From the list of 72 radiation resistant protein candidates, CD44 was identified in the EMT and glycolysis pathways. This study discovered that CD44 is enriched in radiation resistant cells, irrespective of the treatment schedule (that is conventional or hypofractionated treatment). It was confirmed that CD44 expression was increased, and the therapeutic utility of an anti-CD44 blocking antibody in PCa radio-sensitisation was demonstrated. The study of radioresistant PCa has been limited by technical challenges in obtaining prostate samples following relapse (invasive procedure and very little tissue obtainable). As such, the creation of isogenic radioresistant PCa cell lines is one avenue to address this clinical problem. Although our research is primarily based on an isogenic DU145 PCa cell line model, we were able to validate increased CD44 expression in a second isogenic PC3 PCa cell line model, suggesting that our findings are not cell-line specific. Further work investigating the therapeutic role of CD44 in an in vivo model, as well its potential as a predictive biomarker [59] are needed. Additional avenues of research will also focus on those proteins uniquely dysregulated in HF cells, which may contribute to its clinical advantage over CF.

## Supplementary Material

SI Figure 2

SI Figure 3

SI Figure1

SI Table 3

SI Table 2

Si Table 1

## Data Availability

All mass spectrometry raw data has been deposited to the Mass Spectrometry Interactive Virtual Environment (MassIVE) with the following MassiVE ID: MSV000086545 and FTP link: ftp://massive.ucsd.edu/MSV000086545/.

## Clinical relevance

Radiation therapy remains a definitive treatment modality for prostate cancer with curative intent. While it is an effective treatment for a large majority of patients, cancer relapse can occur and is challenging to salvage. Radiation resistance is seen in both conventional and hypofractionated radiation therapy treatment schedules. Potential therapeutic targets to improve radiation remain elusive. This study is the first published proteomic analysis of prostate cancer cell lines generated from clinically relevant treatment schedules. This study uses an isogenic radiation resistant cell line (DU145-CF, DU145-HF, and DU145-PAR) to investigate the proteome and elucidate the pathways and proteins implicated in persistence of prostate cancer following radiation therapy. The cell surface protein CD44 has been implicated in radiation resistance in a range of cancer types and we suggest that it may also represent a potential target in prostate cancer.
