# Supplementary material for: A proteomic investigation of isogenic radiation resistant prostate cancer cell lines
Source: Proteomics Clin Appl. 2021 Jun 30;15(5):2100037. doi: 10.1002/prca.202100037 (PMC8448965; doi:10.1002/prca.202100037)
Supplement: Supplementary file 4 — Supporting information [file PRCA-15-0-s001.pdf]

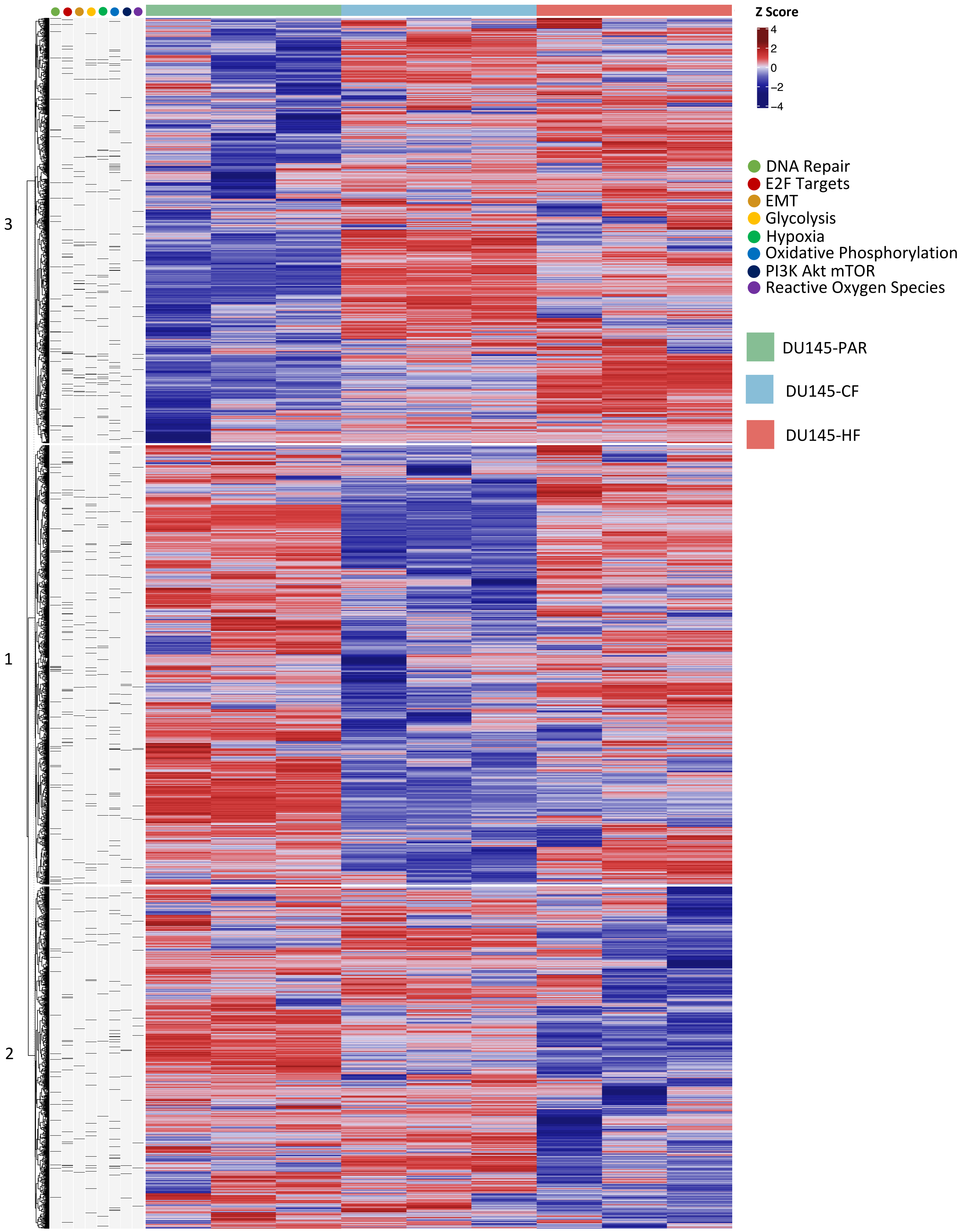

Supplementary Figure 01: Proteomic expression of radiation resistant cell lines.
Heatmap showing the z-scores calculated from the relative protein abundance (log_2 iBAQ values) for each of the 4410 proteins identified in the whole cell lysates for DU145-PAR (green), DU145-CF and DU145-HF (red) cells. Hard clustering was performed on this data with an ideal cluster of three identified. Select gene lists of interest from the Hallmarks of Cancer Gene List from the Molecular Signature Database (MsigDB) was used to create the left side annotation. A simplified version is in Figure 3A.
