# Supplementary material for: A proteomic investigation of isogenic radiation resistant prostate cancer cell lines
Source: Proteomics Clin Appl. 2021 Jun 30;15(5):2100037. doi: 10.1002/prca.202100037 (PMC8448965; doi:10.1002/prca.202100037)
Supplement: Supplementary file 5 — Supporting information [file PRCA-15-0-s003.pdf]

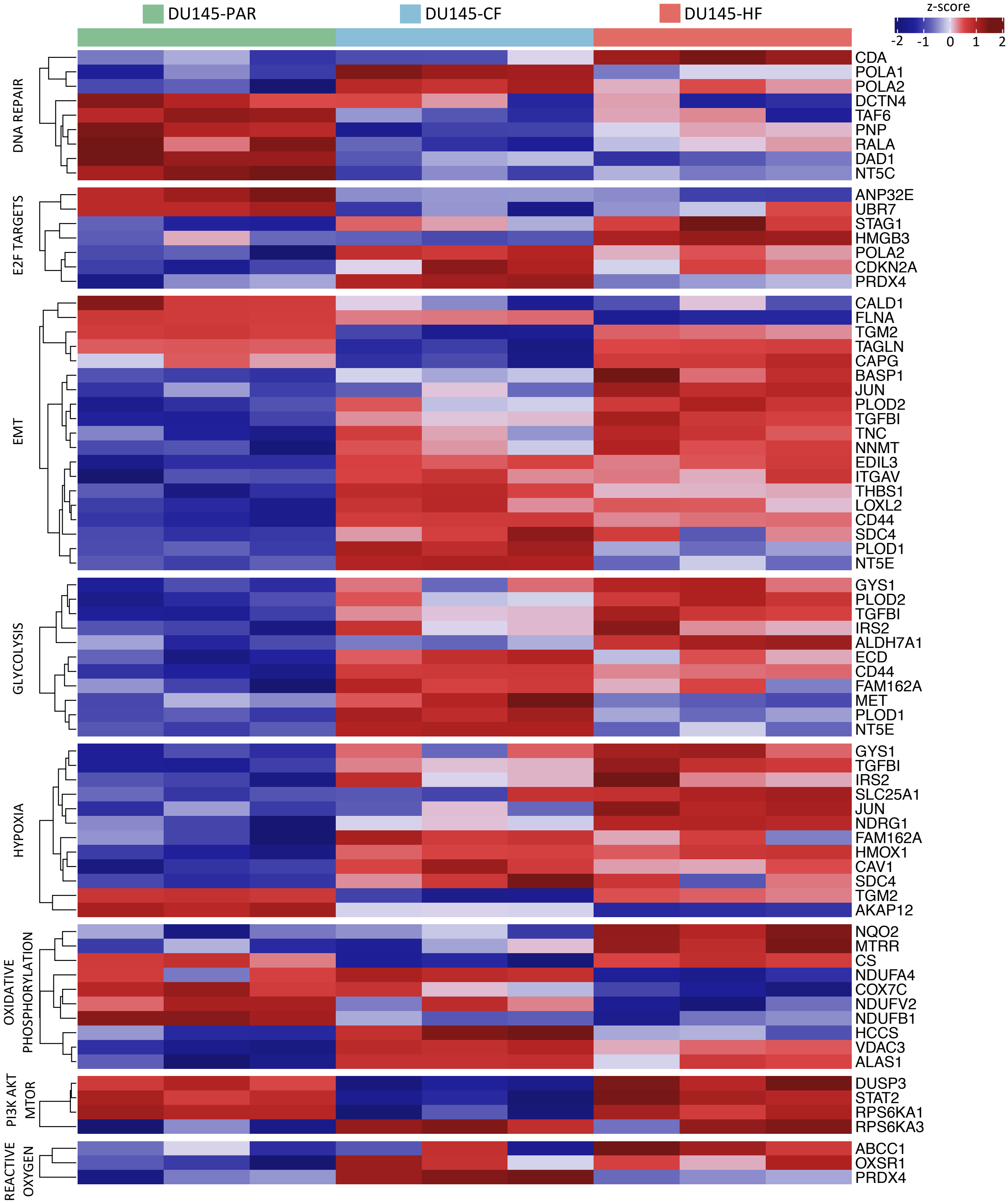

Supplementary Figure 02: Pathways implicated in radiation resistance.
The 4,410 proteins detected in the whole cell lysate data across the DU145-PAR, DU145-CF, and DU145-HF cell lines compared to the 50 Hallmarks of Cancer gene lists from the Molecular Signatures Database (MsigDB). Pathways of interest – which have previously been implicated in radiation resistance and with significantly differentially expressed proteins – showing changes across the three cell lines were selected and are listed on the left of this stacked heatmap. Normalized ibaq values were converted to z-scores for data visualization.
