# Supplementary material for: A proteomic investigation of isogenic radiation resistant prostate cancer cell lines
Source: Proteomics Clin Appl. 2021 Jun 30;15(5):2100037. doi: 10.1002/prca.202100037 (PMC8448965; doi:10.1002/prca.202100037)
Supplement: Supplementary file 6 — Supporting information [file PRCA-15-0-s005.pdf]

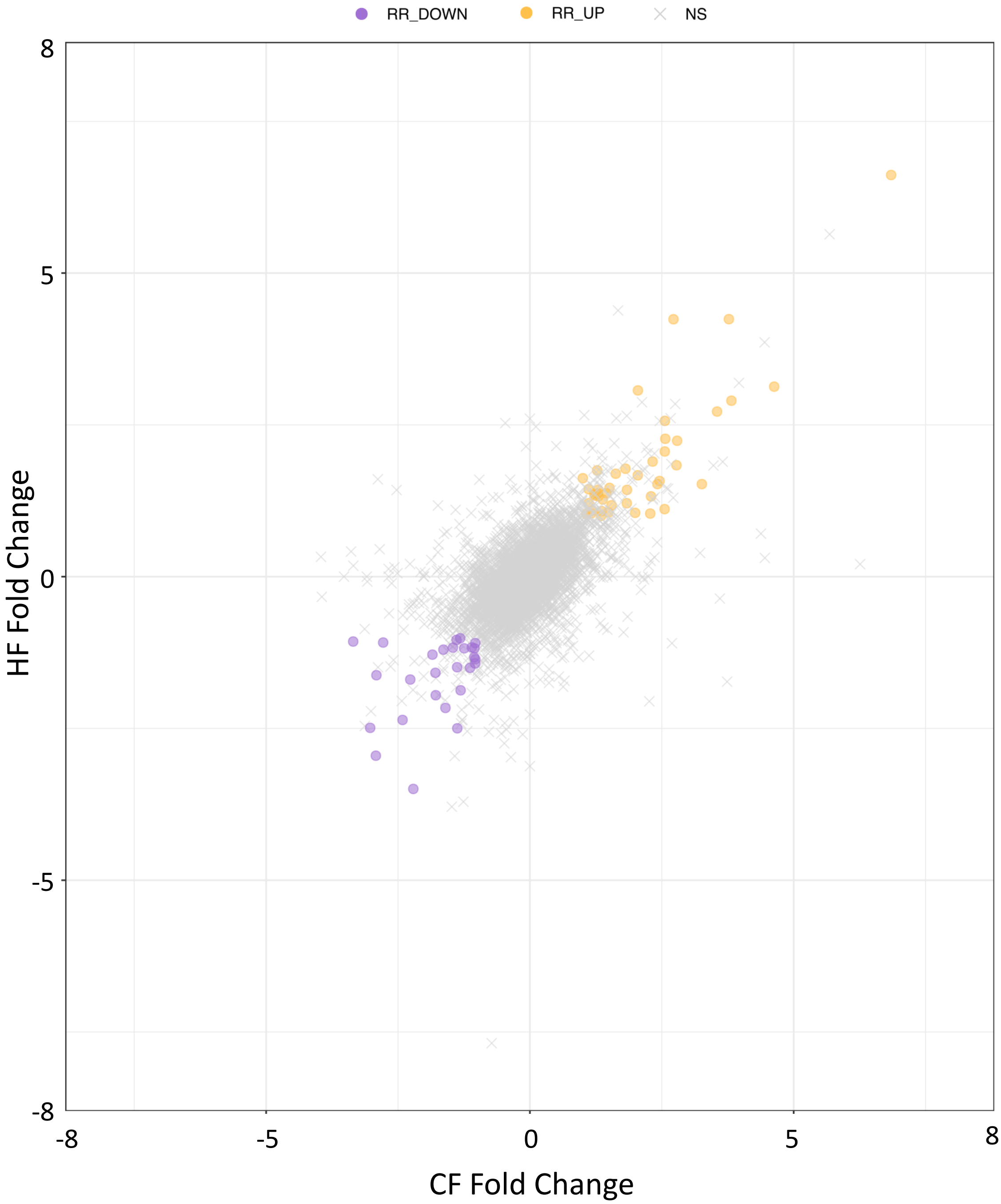

Supplementary Figure 3: Radiation Enriched Proteins.

Scatter plot showing the fold change of the DU145-HF cell lines (as compared to DU145-PAR) and the DU145-CF cells (as compared to DU145-PAR). Circles represent proteins with a p-value < 0.05. Lower left quadrant and upper right quadrant (representing radiation resistant downregulated and upregulated proteins, respectively) are highlight and were used as the input data for Figure 4.A.
